# Attitudes and Expectations of Clinical Research Participants Toward Digital Health and Mobile Dietary Assessment Tools: Cross-Sectional Survey Study

**DOI:** 10.3389/fdgth.2022.794908

**Published:** 2022-03-09

**Authors:** Florent Schäfer, Laurent Quinquis, Maxime Klein, Joséphine Escutnaire, Frédéric Chavanel, Hélène Chevallier, Guy Fagherazzi

**Affiliations:** ^1^Danone Nutricia Research, Palaiseau, France; ^2^UFR Médecine et Pharmacie, Université de Poitiers, Poitiers, France; ^3^Soladis, Lyon, France; ^4^Biofortis, Saint-Herblain, France; ^5^Deep Digital Phenotyping Research Unit, Department of Population Health, Luxembourg Institute of Health, Strassen, Luxembourg

**Keywords:** clinical research, dietary assessment tools, digital health, social media, survey studies, chatbots, clinical operations, patient centricity

## Abstract

**Background:**

The adoption of health technologies is key to empower research participants and collect quality data. However, the acceptance of health technologies is usually evaluated in patients or healthcare practitioners, but not in clinical research participants.

**Methods:**

A 27-item online questionnaire was provided to the 11,695 members of a nutrition clinical research participant database from the Nantes area (France), to assess (1) participants' social and demography parameters, (2) equipment and usage of health apps and devices, (3) expectations in research setting and (4) opinion about the future of clinical research. Each item was described using frequency and percentage overall and by age classes. A global proportion comparison was performed using chi-square or Fisher-exact tests.

**Results:**

A total of 1529 respondents (81.0% women, 19.0% men) completed the survey. Main uses of health apps included physical activity tracking (54.7%, age-related group difference, *p* < 0.001) and food quality assessment (45.7%, unrelated to age groups). Overall, 20.4% of respondents declared owning a connected wristband or watch. Most participants (93.8%) expected the use of connected devices in research. However, protection of personal data (37.5%), reliability (35.5%) and skilled use of devices (28.5%) were perceived as the main barriers. Most participants (93.3%) would agree to track their food intake using a mobile app, and 80.5% would complete it for at least a week while taking part in a clinical study. Only 13.2% would devote more than 10 min per meal to such record. A majority (60.4%) of respondents would accept to share their social media posts in an anonymous way and most (82.2%) of them would accept to interact with a chatbot for research purposes.

**Conclusions:**

Our cross-sectional study suggests that clinical study participants are enthusiastic about all forms of digital health technologies and participant-centered studies but remain concerned about the use of personal data. Repeated assessments are suggested to evaluate the research participant's interest in technologies following the increase in use and demand for innovative health services during the pandemic of COVID-19.

## Graphical Abstract

Attitudes and expectations of clinical research participants towards digital health and mobile dietary assessment tools: Main results of a cross-sectional survey study. 
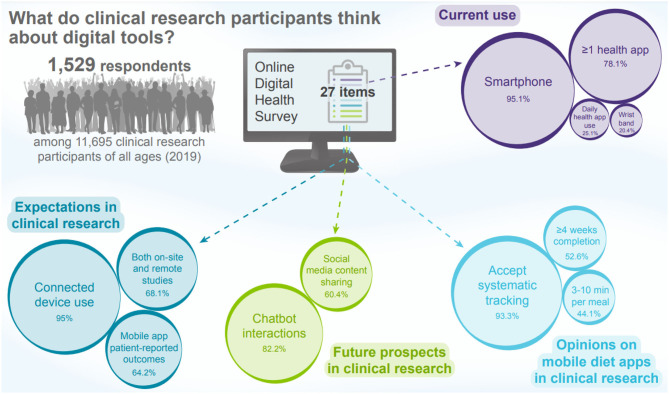

## Introduction

### Patients and Health Care Workers Attitudes Toward Digital Health

There is an increasing use and demand in health technologies going hand in hand with hardware penetration in the global population (1) and the overall automation of daily life, expected to improve society, economy and quality of life (2). While public awareness in digital health is rising (3), attitudes toward health technologies may vary, as some users can be interested in managing appointments and the self-tracking of fitness, diet (4), or vital signs (3, 5) as reflected by recent surveys (3, 4). Recently, patients reported the frequent use of health technologies, including websites (while decreasing), mobile apps, electronic medical records, with a noticeable trend for wearable and smart devices as well as social media (3). Intention to use such technologies has been assessed in samples of patients (6–8), students (9), health workers (10) and in multiple groups at the same time (11–13) to identify demographic or socio-economic determinants of the successful implementation of health technologies. However, these determinants are not usually evaluated in groups of clinical study participants specifically, and it is not known whether the recent trends associated with the use of health technologies are consistent with expectations from research participants in clinical settings.

### The Specific Case of Clinical Research Participants

Volunteering for a clinical study is a personal choice (14), which can be driven by the potential of benefiting personally as well as the possibility to help others (15): Despite the risks associated with the exposure to novel methods and solutions, research participants remain motivated by a desire to contribute to science (16). We can therefore expect that study participants are keen to complete study-specific surveys, monitor their vital signs, or share their health information with investigators. Patient empowerment has been defined by Affinito et al. as “the control of patients over their health and condition, as well as their ability to be more involved in their healthcare” (17). The digital transformation of healthcare allows patients to manage their conditions by facilitating diagnosis, prevention, and treatment (17–19). Therefore, the implementation of mobile technologies in clinical research represents an opportunity to facilitate and streamline the collection of quality data (20), inform clinical care and decision-making (21) while emancipating research participants (22). While participant engagement is getting more attention from sponsors (23), multiple tools may be implemented to collect more data and empower research participants. However, it was reported that the average number of protocol endpoints and procedures recently doubled in <10 years to support secondary and exploratory parameters (24). In a context of clinical research transformation supported by the use of technology, the overuse of tools or the use of inappropriate ones may bear the risks of unnecessary burden and lack of compliance, leading to missing or incorrect data and early dropout (21). Assessing participants' equipment and intention to use tech-enabled health solutions is key to ensuring the scientific validity of research studies. Still, only a few studies recently evaluated how health technologies are perceived among clinical study participants. One study recently evaluated the indicators of retention of a large sample of research participants in remote, digital setting (25). Some initiatives, like the Trial Feedback Questionnaire (26) were developed and may be used to gather participant feedbacks on the tools that are used in clinical studies. However, this questionnaire can only be used in a single indication or study at a time and after completion, preventing a proactive implementation in research settings (27). Meanwhile, quality and relevance of research can be improved by patient and public involvement in research, which is not systematically considered in research protocols (28).

### Survey Objectives

As clinical research participants are conclusively affected by study designs and by the implementation of technologies (28), we considered them as our survey study population. Our objective was to evaluate their attitudes and expectations toward digital patient-generated health data and food tracking mobile apps and understand if their choices are associated with age groups.

## Methods

### Survey Design

Surveys are a cost-effective and non-interventional option to collect qualitative and semi-quantitative information to enable further research (29). An original survey was designed to understand expectations and concerns of clinical research participants toward digital health. A brief introduction was provided to describe the aims of the survey. The first section was designed to collect social and demography parameters. As research participants often have the possibility of bringing their own pocket-sized solutions instead of being provided with study-specific hardware, it was decided to investigate their equipment and their expectations in clinical research separately. Current equipment was assessed in a second section, while expectations in research settings were investigated in a third one. Furthermore, the appeal for rather advanced technologies which are not commonly used, such as sharing of social media posts and interactions with chatbots, is not frequently evaluated. As the sharing of such information can be perceived as controversial, those items were explored separately at the end of the questionnaire and was the purpose of a fourth section. Due to the limited published work investigating research participants interest and expectations toward in digital health, main items were designed to understand appeal for popular technologies (3–5), and understand concerns raised by their implementation in research. The use of free text was avoided as much as possible to focus on qualitative modalities. In this study, we referred to “connected devices” when assessing participant's interest in wearable and non-wearable smart health monitoring solutions.

Items of the survey were provided to members of the personnel affiliated to the sponsor and investigator to gather feedback during its design phase. The list of items was eventually narrowed down to a 27-item multiple-choice questionnaire (Additional File 1). To avoid underestimation of both expectations and especially concerns associated with the use of digital tools, it was decided not to limit the number of answers when selecting the modalities. Questions were formulated to limit ambiguity as much as possible.

### Recruitment of Respondents

No health or personal data was collected as the survey items were designed to avoid the collection of sensitive or personally identifiable information. Participants were considered eligible to participate in this survey study if they were 18 years old or older, and currently screened or enrolled in a clinical study. Former clinical research participants were also considered eligible. No quota-based sampling was performed. A total of 11,695 members (8,386 women, 3,309 men) of a clinical research participant database (owned by *Biofortis*) from the Nantes region in France were contacted by email in May 2019 to read an information sheet and complete this anonymous questionnaire. The entire panel was contacted. The participating site, specialized in the conduct of nutrition clinical studies, was selected based on the research team's interest in the survey study and their ability to recruit a large sample of respondents from various age ranges and conditions, including healthy volunteers.

Participants completed an online version of the questionnaire hosted on Microsoft Forms (30) which allowed them to remain anonymous. No individually identifiable information or health data was collected, and no risk associated with data privacy was identified. Following a legal opinion from an independent expert consultant, this survey was not considered a clinical study requiring approval from an independent ethics committee. The survey was available online from the 23rd of May 2019 to the 1st of July 2019. No reminders were sent during this period.

### Statistical Analyses

Each item of the survey was described using frequency and percentage overall and by age classes. For each item, a global proportion comparison was performed between the age classes using the chi-square test, or the Fisher-exact test if any expected cell count was inferior to 5 as an alternative to the chi-square test. In case of global effect, pairwise proportion comparisons between age classes were completed using chi-square test or Fisher-exact test accordingly. The results of comparisons were adjusted to account for multiple comparisons according to the Bonferroni-Holm method. All analyses were performed using SAS v9.4®. A margin error of 5% was used for statistical tests.

## Results

### Study Participants

**Figure 1:**
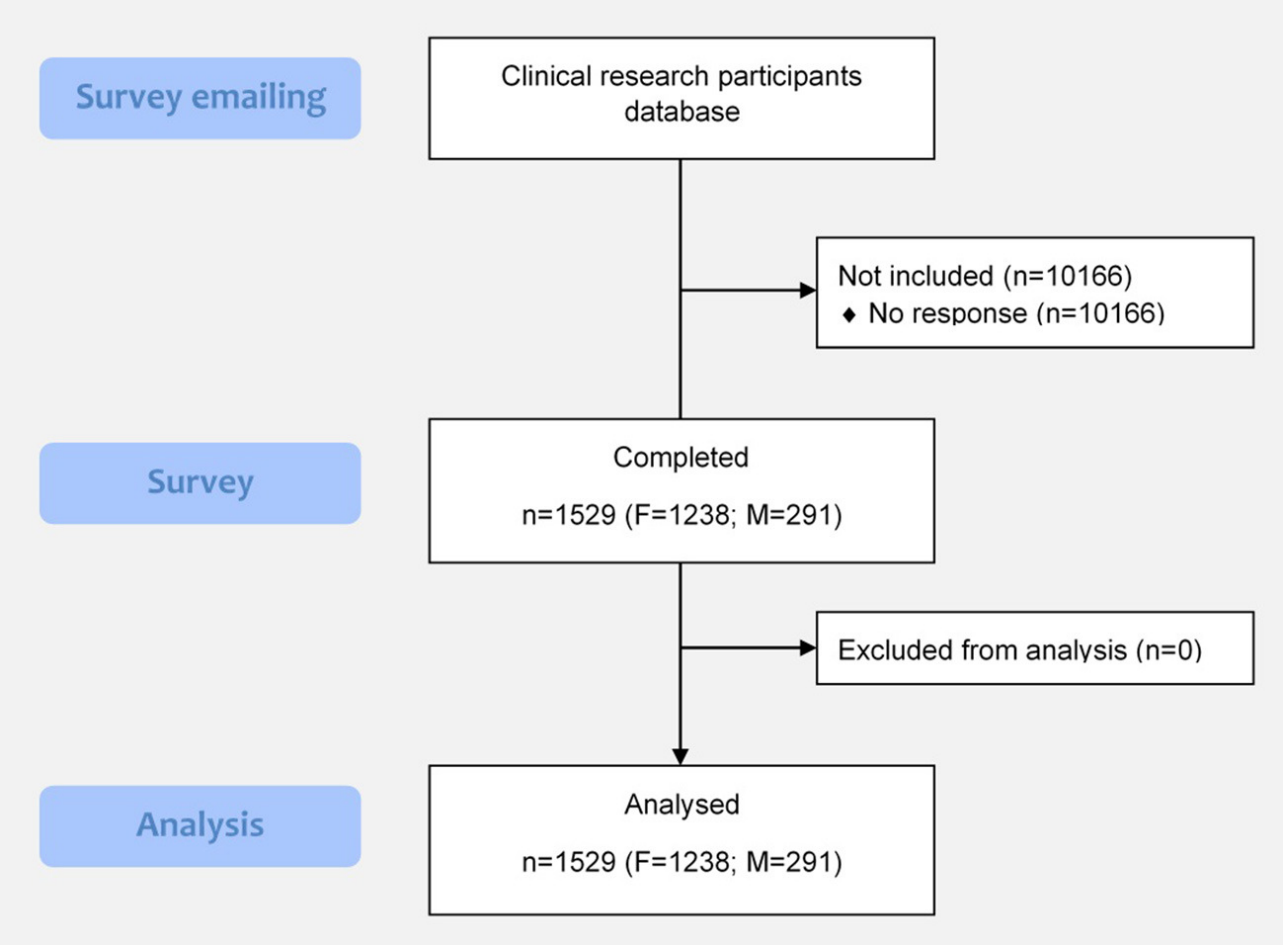
Flow chart.

All 11,695 members of the clinical research participants database (8,386 women, 3,309 men) were contacted to complete our survey. Among the 1,529 respondents who completed the survey, 81.0% were female (Figure 1). A share of 14.77% of women accepted to complete the survey (1239/8386), while 8.76% of men (290/3309) completed it. Most of respondents were aged 25–54 years old (64.9%) and lived in medium-size (10,000–50,000 inhabitants) and large cities (>50,000 inhabitants) (28.6 and 40.0%, respectively). While 35.4% of them had received primary education, 15.0% reached third level education. However, 11.1% declared having not received any formal education (Table 1).

**Table 1:** Demography of respondents.

**Age class (years)**	**Total *N* = 1,529**	**18–24**	**25–34**	**35–44**	**45–54**	**55–64**	**≥65**
Number of respondents	–	102 (6.7%)	337 (22.0%)	350 (22.9%)	306 (20.0%)	265 (17.3%)	169 (11.1%)
**Sex**							
Female	1,238 (81.0%)	88 (86.3%)	274 (81.3%)	290 (82.9%)	243 (79.4%)	211 (79.6%)	132 (78.1%)
Male	291 (19.0%)	14 (13.7%)	63 (18.7%)	60 (17.1%)	63 (20.6%)	54 (20.4%)	37 (21.9%)
**Place of residence**							
Large city (over 50,000 inhabitants)	612 (40.0%)	57 (55.9%)	137 (40.7%)	117 (33.4%)	122 (39.9%)	112 (42.3%)	67 (39.6%)
Medium-sized city (10,000–50,000 inhabitants)	438 (28.6%)	28 (27.5%)	105 (31.2%)	98 (28.0%)	83 (27.1%)	76 (28.7%)	48 (28.4%)
Small city (2,000–10,000 inhabitants)	380 (24.9%)	15 (14.7%)	78 (23.1%)	106 (30.3%)	79 (25.8%)	61 (23.0%)	41 (24.3%)
Village (<2,000 inhabitants)	99 (6.5%)	2 (2.0%)	17 (5.0%)	29 (8.3%)	22 (7.2%)	16 (6.0%)	13 (7.7%)
**Level of education**							
Primary education	541 (35.4%)	31 (30.4%)	109 (32.3%)	120 (34.3%)	93 (30.4%)	103 (38.9%)	85 (50.3%)
Lower secondary education	372 (24.3%)	16 (15.7%)	73 (21.7%)	91 (26.0%)	97 (31.7%)	65 (24.5%)	30 (17.8%)
Secondary education	216 (14.1%)	28 (27.5%)	58 (17.2%)	47 (13.4%)	29 (9.5%)	38 (14.3%)	16 (9.5%)
Third level	230 (15.0%)	22 (21.6%)	63 (18.7%)	68 (19.4%)	42 (13.7%)	24 (9.1%)	11 (6.5%)
No formal education	170 (11.1%)	5 (4.9%)	34 (10.1%)	24 (6.9%)	45 (14.7%)	35 (13.2%)	27 (16.0%)

### Smartphone Use, Expectations and Concerns

Most (95.1%) reported owning a smartphone, with the younger age-classes (18–44 years; 100% in the 18–24, 98.8% in the 25–34 and 98.8% in the 35–44) showing a marked difference with the oldest (45 and over; 93.8% in the 45–54, 91.7% in the 55–64 and 84.6% in the 65 and over) (*p* < 0.001).

Regarding the use of health-related mobile apps, 78.1% of participants declared using at least one of them and 54.6% declared using two or more of them. Statistically significant differences were observed between age groups (*p* < 0.001): 71.6% of the participants aged 18–24 and 64.5% aged 25–34 declared using at least two health-related mobile apps whereas they were 45.3% aged 55–64 and 32.2% aged 65 or over to report the same use. However, there was no between-age-groups difference regarding neither the frequency of use of such apps (*p* = 0.093) with a majority of participants (76.2%) using them at least once a week, nor the duration of use (*p* = 0.376) with a majority of participants declaring a duration of 1–5 min per use (56.6%).

The main expectation toward such apps was the ability to monitor their physical activity (54.7%) which was more prevalent (*p* < 0.001) in the participants up to 44 years (ranging from 57.6 to 63.7%) as compared to the oldest ones from 55 and over (ranging from 43.2 to 53.6%). The ability to assess food quality arrived second (45.7%) without between-age-group difference. About one third of the study participants pointed out weight loss monitoring, with a preference for the individuals up to 44 years (*p* < 0.001). Other marked expectations included maintaining/improving their health and sleep monitoring. The latter was age dependent (*p* < 0.001) as it appeared particularly important for the youngest participants (51% of the 18–24 years old) and not that much for the ones aged 65 and over (17.2%). Interestingly, few (7.8%) declared having no expectation, but the feature was more present in the oldest classes (*p* < 0.001). Details about other expectations are provided in Table 2.

**Table 2:** Smartphone use by age class.

	**Total**	**18–24 years**	**25–34 years**	**35–44 years**	**45–54 years**	**55–64 years**	**65 years or over**	**Global *p*-value**
		**A**	**B**	**C**	**D**	**E**	**F**	
**Do you own a smartphone?**								
	***N*** **= 1,529**	***N*** **= 102**	***N*** **= 337**	***N*** **= 350**	***N*** **= 306**	***N*** **= 265**	***N*** **= 169**	
No	75 (4.9%)	0 (0.0%)	4 (1.2%)	4 (1.1%)	19 (6.2%)	22 (8.3%)	26 (15.4%)	**<0.001**
Yes	1454 (95.1%)	102 (100.0%)	333 (98.8%)	346 (98.9%)	287 (93.8%)	243 (91.7%)	143 (84.6%)	
Significant difference with other age class	–	***D E F***	***D E F***	***D E F***	***A B C F***	***A B C***	***A B C D***	
**If yes, how many health-related mobile apps do you use? (Nutrition, physical activity, weight, sleep, health coaching, well-being, meditation, etc.)**								
	***N*** **= 1,454**	***N*** **= 102**	***N*** **= 333**	***N*** **= 346**	***N*** **= 287**	***N*** **= 243**	***N*** **= 143**	
None	318 (21.9%)	9 (8.8%)	42 (12.6%)	63 (18.2%)	70 (24.4%)	75 (30.9%)	59 (41.3%)	**<0.001**
One	341 (23.5%)	20 (19.6%)	76 (22.8%)	84 (24.3%)	65 (22.6%)	58 (23.9%)	38 (26.6%)	
Two or three	614 (42.2%)	51 (50.0%)	162 (48.6%)	154 (44.5%)	122 (42.5%)	87 (35.8%)	38 (26.6%)	
Four or more	181 (12.4%)	22 (21.6%)	53 (15.9%)	45 (13.0%)	30 (10.5%)	23 (9.5%)	8 (5.6%)	
Significant difference with other age class	–	***D E F***	***D E F***	***E F***	***A B F***	***A B C***	***A B C D***	
**If you use at least one health-related mobile app, please specify how often you use it**								
	***N*** **= 1,136**	***N*** **= 93**	***N*** **= 291**	***N*** **= 283**	***N*** **= 217**	***N*** **= 168**	***N*** **= 84**	
Less than once a month	83 (7.3%)	5 (5.4%)	22 (7.6%)	18 (6.4%)	20 (9.2%)	14 (8.3%)	4 (4.8%)	0.093
1–3 times a month	188 (16.5%)	23 (24.7%)	46 (15.8%)	41 (14.5%)	43 (19.8%)	16 (9.5%)	19 (22.6%)	
Once a week	243 (21.4%)	18 (19.4%)	69 (23.7%)	59 (20.8%)	35 (16.1%)	39 (23.2%)	23 (27.4%)	
2–5 times a week	337 (29.7%)	31 (33.3%)	83 (28.5%)	85 (30.0%)	66 (30.4%)	49 (29.2%)	23 (27.4%)	
At least once a day	285 (25.1%)	16 (17.2%)	71 (24.4%)	80 (28.3%)	53 (24.4%)	50 (29.8%)	15 (17.9%)	
Significant difference with other age class	–	–	–	–	–	–	–	
**On average, how much time do you spend on these health apps per use?**								
	***N*** **= 1,136**	***N*** **= 93**	***N*** **= 291**	***N*** **= 283**	***N*** **= 217**	***N*** **= 168**	***N*** **= 84**	
<1 min	123 (10.8%)	11 (11.8%)	28 (9.6%)	26 (9.2%)	24 (11.1%)	28 (16.7%)	6 (7.1%)	0.376
1–5 min	643 (56.6%)	55 (59.1%)	165 (56.7%)	168 (59.4%)	115 (53.0%)	91 (54.2%)	49 (58.3%)	
Over 5 min	370 (32.6%)	27 (29.0%)	98 (33.7%)	89 (31.4%)	78 (35.9%)	49 (29.2%)	29 (34.5%)	
Significant difference with other age class	–	–	–	–	–	–	–	

*Global p-value, when significant; Age classes, Significant difference with other age classes; N = number of participants per age class*.

The most frequently expressed concern toward such apps was the frequency of advertisements (46.6%) irrespective of the age class of the respondents. Data protection (37.0%) and lack of reliability (23.7%) were also frequently expressed, both showing a difference in prevalence between age groups (*p* < 0.001 and *p* = 0.002, respectively). The youngest (18–24 years) participants were less concerned about personal data protection and more about reliability issues than their older counterparts. Details about other perceived issues are provided in Table 3.

**Table 3:** Smartphone apps expectations and concerns by age class.

	**Total**	**18–24 years**	**25–34 years**	**35–44 years**	**45–54 years**	**55–64 years**	**65 years or over**	**Global *p*-value**
		**A**	**B**	**C**	**D**	**E**	**F**	
**What are your main expectations from mobile health apps?**								
	***N*** **= 1,529**	***N*** **= 102**	***N*** **= 337**	***N*** **= 350**	***N*** **= 306**	***N*** **= 265**	***N*** **= 169**	
Assessing of your physical activity	836 (54.7%)	65 (63.7%)	194 (57.6%)	218 (62.3%)	164 (53.6%)	122 (46.0%)	73 (43.2%)	**<0.001**
Significant difference with other age class	–	***E F***	***E F***	***E F***	–	***A B C***	***A B C***	
Monitoring your energy intake	430 (28.1%)	34 (33.3%)	95 (28.2%)	112 (32.0%)	83 (27.1%)	68 (25.7%)	38 (22.5%)	0.181
Significant difference with other age class	–	–	–	–	–	–	–	
Weight loss monitoring	577 (37.7%)	49 (48.0%)	154 (45.7%)	140 (40.0%)	116 (37.9%)	76 (28.7%)	42 (24.9%)	**<0.001**
Significant difference with other age class	–	***E F***	***E F***	***E F***	***F***	***A B C***	***A B C D***	
Maintaining/improving your health	502 (32.8%)	35 (34.3%)	102 (30.3%)	116 (33.1%)	107 (35.0%)	92 (34.7%)	50 (29.6%)	0.702
Significant difference with other age class	–	–	–	–	–	–	–	
Monitoring your sleep	474 (31.0%)	52 (51.0%)	115 (34.1%)	119 (34.0%)	94 (30.7%)	65 (24.5%)	29 (17.2%)	**<0.001**
Significant difference with other age class	–	***B C D E F***	***A F***	***A F***	***A F***	***A***	***A B C D***	
Assessing the quality of your food	698 (45.7%)	50 (49.0%)	151 (44.8%)	168 (48.0%)	140 (45.8%)	127 (47.9%)	62 (36.7%)	0.195
Significant difference with other age class	–	–	–	–	–	–	–	
A meditation tool	272 (17.8%)	22 (21.6%)	62 (18.4%)	66 (18.9%)	55 (18.0%)	47 (17.7%)	20 (11.8%)	0.362
Significant difference with other age class	–	–	–	–	–	–	–	
Weight maintenance monitoring	240 (15.7%)	12 (11.8%)	50 (14.8%)	61 (17.4%)	42 (13.7%)	47 (17.7%)	28 (16.6%)	0.549
Significant difference with other age class	–	–	–	–	–	–	–	
Period or pregnancy tracking	216 (14.1%)	48 (47.1%)	92 (27.3%)	64 (18.3%)	12 (3.9%)	0 (0.0%)	0 (0.0%)	**<0.001**
Significant difference with other age class	–	***B C D E F***	***A C D E F***	***A B D E F***	***A B C E F***	***A B C D***	***A B C D***	
No expectations	120 (7.8%)	4 (3.9%)	17 (5.0%)	8 (2.3%)	27 (8.8%)	34 (12.8%)	30 (17.8%)	**<0.001**
Significant difference with other age class	–	***F***	***E F***	***D E F***	***C F***	***B C***	***A B C D***	
Monitoring a chronic condition	82 (5.4%)	4 (3.9%)	15 (4.5%)	19 (5.4%)	23 (7.5%)	15 (5.7%)	6 (3.6%)	0.427
Significant difference with other age class	–	–	–	–	–	–	–	
Weight gain monitoring	81 (5.3%)	8 (7.8%)	17 (5.0%)	25 (7.1%)	15 (4.9%)	8 (3.0%)	8 (4.7%)	0.245
Significant difference with other age class	–	–	–	–	–	–	–	
**What are your main concerns about using health–related mobile apps?**								
	***N*** **= 1,529**	***N*** **= 102**	***N*** **= 337**	***N*** **= 350**	***N*** **= 306**	***N*** **= 265**	***N*** **= 169**	
The frequency of adverts	713 (46.6%)	56 (54.9%)	154 (45.7%)	171 (48.9%)	142 (46.4%)	119 (44.9%)	71 (42.0%)	0.370
Significant difference with other age class	–	–	–	–	–	–	–	
Protection of your personal data	566 (37.0%)	22 (21.6%)	112 (33.2%)	140 (40.0%)	127 (41.5%)	94 (35.5%)	71 (42.0%)	**0.002**
Significant difference with other age class	–	***C D F***	–	***A***	***A***	–	***A***	
Lack of reliability	362 (23.7%)	37 (36.3%)	118 (35.0%)	77 (22.0%)	65 (21.2%)	43 (16.2%)	22 (13.0%)	**<0.001**
Significant difference with other age class	–	***C D E F***	***C D E F***	***A B***	***A B***	***A B***	***A B***	
Location tracking	311 (20.3%)	19 (18.6%)	66 (19.6%)	91 (26.0%)	60 (19.6%)	46 (17.4%)	29 (17.2%)	0.080
Significant difference with other age class	–	–	–	–	–	–	–	
The need to create a personal account	282 (18.4%)	20 (19.6%)	64 (19.0%)	66 (18.9%)	45 (14.7%)	54 (20.4%)	33 (19.5%)	0.574
Significant difference with other age class	–	–	–	–	–	–	–	
Too time–consuming	251 (16.4%)	17 (16.7%)	50 (14.8%)	57 (16.3%)	55 (18.0%)	55 (20.8%)	17 (10.1%)	0.082
Significant difference with other age class	–	–	–	–	–	–	–	
Poorly designed interface	220 (14.4%)	20 (19.6%)	60 (17.8%)	58 (16.6%)	41 (13.4%)	35 (13.2%)	6 (3.6%)	**<0.001**
Significant difference with other age class	–	***F***	***F***	***F***	***F***	***F***	***A B C D E***	
Does not meet your needs	201 (13.1%)	26 (25.5%)	48 (14.2%)	36 (10.3%)	38 (12.4%)	36 (13.6%)	17 (10.1%)	**0.003**
Significant difference with other age class	–	***C D F***	–	***A***	***A***	–	***A***	
Not useful	160 (10.5%)	14 (13.7%)	38 (11.3%)	30 (8.6%)	29 (9.5%)	27 (10.2%)	22 (13.0%)	0.514
Significant difference with other age class	–	–	–	–	–	–	–	

*Global p-value, when significant; Age classes, Significant difference with other age classes; N = number of participants per age class*.

### Connected Watch/Wristband Use, Expectations and Concerns

Among participants to the survey, 20.4% declared using a connected watch or wristband, with a significant between-group difference (*p* = 0.050). Most of them declared using them for assessing physical activity (76.9%), monitoring sleep (39.4%), during sport (37.2%), for cardiovascular monitoring (32.4%) and receiving smartphone notifications (29.5%). Distribution of these uses were not different among the age classes of the participants, except for the latter (*p* = 0.008) for which respondents aged 65 years and over had no interest (4.3%).

The most frequent concerns regarding this type of device were concerns toward their price (46.2%) which was significantly more reported in the youngest classes than in the oldest (*p* < 0.001), and the lack of perceived usefulness (30.8%) which was more often reported in the oldest classes (*p* < 0.001). Results per age group and between-group differences are detailed in Table 4.

**Table 4:** Connected watch/wristband use, expectations and concerns by age class.

	**Total**	**18–24 years**	**25–34 years**	**35–44 years**	**45–54 years**	**55–64 years**	**65 years or over**	**Global *p*-value**
		**A**	**B**	**C**	**D**	**E**	**F**	
**Do you use a connected watch or wristband?**								
	***N*** **= 1,529**	***N*** **= 102**	***N*** **= 337**	***N*** **= 350**	***N*** **= 306**	***N*** **= 265**	***N*** **= 169**	
No	1,217 (79.6%)	86 (84.3%)	266 (78.9%)	266 (76.0%)	236 (77.1%)	217 (81.9%)	146 (86.4%)	**0.050**
Yes	312 (20.4%)	16 (15.7%)	71 (21.1%)	84 (24.0%)	70 (22.9%)	48 (18.1%)	23 (13.6%)	
Significant difference with other age class	–	–	–	–	–	–	–	
**If yes, why?**								
	***N*** **= 312**	***N*** **= 16**	***N*** **= 71**	***N*** **= 84**	***N*** **= 70**	***N*** **= 48**	***N*** **= 23**	
Assessing of your physical activity	240 (76.9%)	12 (75.0%)	56 (78.9%)	67 (79.8%)	59 (84.3%)	32 (66.7%)	14 (60.9%)	0.114
Significant difference with other age class	–	–	–	–	–	–	–	
Monitoring your sleep	123 (39.4%)	6 (37.5%)	32 (45.1%)	32 (38.1%)	32 (45.7%)	18 (37.5%)	3 (13.0%)	0.110
Significant difference with other age class	–	–	–	–	–	–	–	
Sports reasons	116 (37.2%)	5 (31.3%)	35 (49.3%)	34 (40.5%)	23 (32.9%)	15 (31.3%)	4 (17.4%)	0.069
Significant difference with other age class	–	–	–	–	–	–	–	
Cardiovascular monitoring	101 (32.4%)	6 (37.5%)	29 (40.8%)	18 (21.4%)	24 (34.3%)	19 (39.6%)	5 (21.7%)	0.089
Significant difference with other age class	–	–	–	–	–	–	–	
To receive smartphone notifications	92 (29.5%)	6 (37.5%)	31 (43.7%)	23 (27.4%)	20 (28.6%)	11 (22.9%)	1 (4.3%)	**0.008**
Significant difference with other age class	–	–	***F***	–	–	–	***B***	
Weight loss monitoring	69 (22.1%)	4 (25.0%)	20 (28.2%)	19 (22.6%)	17 (24.3%)	5 (10.4%)	4 (17.4%)	0.316
Significant difference with other age class	–	–	–	–	–	–	–	
Improving/maintaining your health	65 (20.8%)	2 (12.5%)	17 (23.9%)	18 (21.4%)	20 (28.6%)	8 (16.7%)	0 (0.0%)	0.070
Significant difference with other age class	–	–	–	–	–	–	–	
Because of the device's appearance	34 (10.9%)	1 (6.3%)	10 (14.1%)	11 (13.1%)	7 (10.0%)	4 (8.3%)	1 (4.3%)	0.713
Significant difference with other age class	–	–	–	–	–	–	–	
Monitoring a chronic condition	2 (0.6%)	0 (0.0%)	1 (1.4%)	0 (0.0%)	1 (1.4%)	0 (0.0%)	0 (0.0%)	0.756
Significant difference with other age class	–	–	–	–	–	–	–	
**What concerns you about using a connected watch or wristband?**								
	***N*** **= 1,529**	***N*** **= 102**	***N*** **= 337**	***N*** **= 350**	***N*** **= 306**	***N*** **= 265**	***N*** **= 169**	
Price	707 (46.2%)	73 (71.6%)	183 (54.3%)	174 (49.7%)	130 (42.5%)	92 (34.7%)	55 (32.5%)	**<0.001**
Significant difference with other age class	–	***B C D E F***	***A D E F***	***A E F***	***A B***	***A B C***	***A B C***	
Not useful	471 (30.8%)	35 (34.3%)	93 (27.6%)	79 (22.6%)	88 (28.8%)	100 (37.7%)	76 (45.0%)	**<0.001**
Significant difference with other age class	–	–	***F***	***E F***	***F***	***C***	***B C D***	
Appearance	262 (17.1%)	18 (17.6%)	61 (18.1%)	70 (20.0%)	48 (15.7%)	47 (17.7%)	18 (10.7%)	0.168
Significant difference with other age class	–	–	–	–	–	–	–	
Protection of your personal data	249 (16.3%)	11 (10.8%)	43 (12.8%)	57 (16.3%)	50 (16.3%)	56 (21.1%)	32 (18.9%)	0.056
Significant difference with other age class	–	–	–	–	–	–	–	
Discomfort	237 (15.5%)	15 (14.7%)	57 (16.9%)	65 (18.6%)	59 (19.3%)	20 (7.5%)	21(12.4%)	**0.001**
Significant difference with other age class	–	–	***E***	***E***	***E***	***B C D***	–	
Location tracking	197 (12.9%)	8 (7.8%)	41 (12.2%)	49 (14.0%)	41 (13.4%)	38 (14.3%)	20 (11.8%)	0.610
Significant difference with other age class	–	–	–	–	–	–	–	
Battery life	194 (12.7%)	15 (14.7%)	49 (14.5%)	48 (13.7%)	35 (11.4%)	33 (12.5%)	14 (8.3%)	0.397
Significant difference with other age class	–	–	–	–	–	–	–	
Weight	87 (5.7%)	5 (4.9%)	18 (5.3%)	24 (6.9%)	17 (5.6%)	15 (5.7%)	8 (4.7%)	0.926
Significant difference with other age class	–	–	–	–	–	–	–	

*Global p-value, when significant; Age classes, Significant difference with other age classes; N = number of participants per age class*.

### Expectations and Concerns Regarding These Connected Devices in Clinical Research

**Figure 2:**
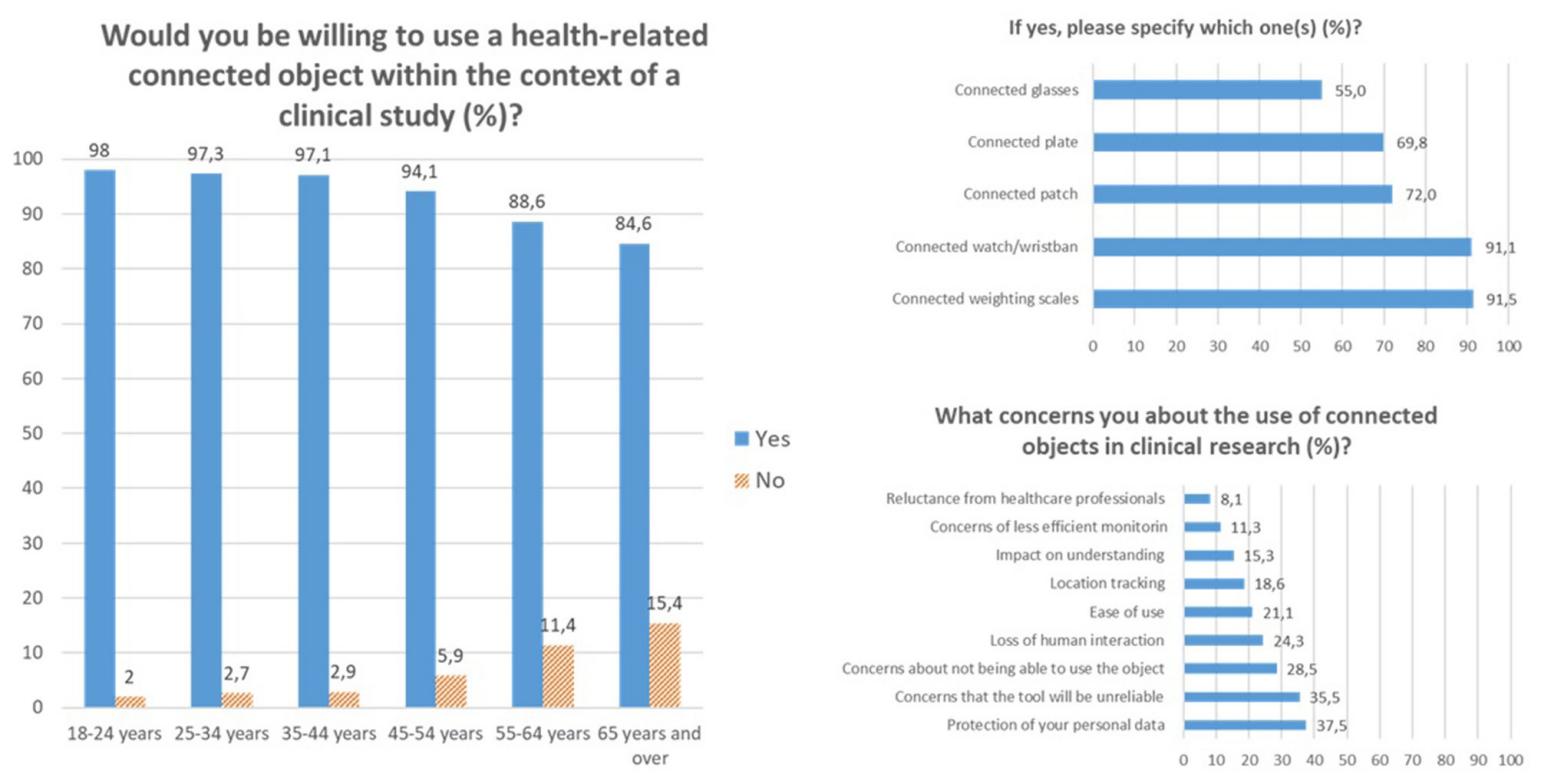
Expectations and concerns related to the use of health-related connected devices in medical research.

In the context of clinical research, 93.8% of participants were favorable to the use of connected (smart) objects with between-group differences (*p* < 0.001) with the youngest classes being more often inclined to use connected devices, irrespective of the type of device (*p* < 0.001 for weighting scales, smartwatches/wristbands, patches, plates and glasses). In the context of clinical research, the protection of personal data (37.5%) remained the main concern with the youngest class (18–24 years old) being significantly less often concerned than the oldest (*p* < 0.001). Other concerns included the reliability (35.5%) and the ability to use the device (28.5%), the former being more reported by the youngest study population (*p* < 0.001) and the latter by the oldest one (55 years and over, *p* > 0.001). These classes also more often dreaded the loss of human interaction and expressed more frequently concerns about the ease of use than the younger classes (*p* < 0.001 and *p* = 0.009 respectively). These results are presented in Figure 2.

During their participation in a clinical research, participants would mostly prefer having both on-site and remote conduct of a clinical study (68.1%), the oldest class preferring more often exclusive physical setting than the youngest (*p* < 0.001). The same significant tendency (*p* < 0.001) was noted regarding the best way to provide information regarding a clinical research protocol. Overall, respondents preferred a fun mobile app (50.2%) rather than paper explanation (25.0%) or video support and discussion with a professional (24.7%). The oldest class (65 years old and over) preferred paper explanations (43.8%) to mobile apps (36.7%). Regarding the way to report health-related data, mobile app questionnaire was preferred (64.2%) to computer questionnaire (29.0%) and paper questionnaire (6.8%) except in the oldest group in which computer questionnaire was preferred (58.6%) over the former (between age group difference: *p* < 0.001). Six out of ten participants would agree to share in a secure and anonymous system their social media content with research staff with significant difference between age groups (*p* < 0.001). Indeed, less than half of the respondents aged 65 years and over were inclined to share this content. Finally, 82.2% of participants would agree to interact with a chatbot and send their data to a member of the research team with notably the oldest class being less likely to agree than the others (*p* < 0.001). Results and responses per age group and between-group differences are detailed in Table 5.

**Table 5:** Use of connected devices and social media enabled features in clinical research by age class.

	**Total**	**18–24 years**	**25–34 years**	**35–44 years**	**45–54 years**	**55–64 years**	**65 years or over**	**Global *p*-value**
		**A**	**B**	**C**	**D**	**E**	**F**	
**Would you be willing to use a health-related connected object within the context of a clinical study?**								
	***N*** **= 1529**	***N*** **= 102**	***N*** **= 337**	***N*** **= 350**	***N*** **= 306**	***N*** **= 265**	***N*** **= 169**	
No	95 (6.2%)	2 (2.0%)	9 (2.7%)	10 (2.9%)	18 (5.9%)	30 (11.3%)	26 (15.4%)	**<0.001**
Yes	1434 (93.8%)	100 (98.0%)	328 (97.3%)	340 (97.1%)	288 (94.1%)	235 (88.7%)	143 (84.6%)	
Significant difference with other age class	–	***E F***	***E F***	***E F***	***F***	***A B C***	***A B C D***	
**If yes, please specify which one(s)**								
	***N*** **= 1434**	***N*** **= 100**	***N*** **= 328**	***N*** **= 340**	***N*** **= 288**	***N*** **= 235**	***N*** **= 143**	
Connected weighing scales	1312 (91.5%)	92 (92.0%)	317 (96.6%)	319 (93.8%)	263 (91.3%)	204 (86.8%)	117 (81.8%)	**<0.001**
Significant difference with other age class	–	–	***D E F***	***E F***	***B F***	***B C***	***B C D***	
Connected watch/wristband	1306 (91.1%)	94 (94.0%)	311 (94.8%)	323 (95.0%)	262 (91.0%)	208 (88.5%)	108 (75.5%)	**<0.001**
Significant difference with other age class	–	***F***	***F***	***E F***	***F***	***C F***	***A B C D E***	
Connected patch	1,032 (72.0%)	77 (77.0%)	275 (83.8%)	254 (74.7%)	201 (69.8%)	147 (62.6%)	78 (54.5%)	**<0.001**
Significant difference with other age class	–	***F***	***C D E F***	***B E F***	***B F***	***B C***	***A B C D***	
Connected plate	1001 (69.8%)	79 (79.0%)	260 (79.3%)	251 (73.8%)	199 (69.1%)	141 (60.0%)	71 (49.7%)	**<0.001**
Significant difference with other age class	–	***E F***	***D E F***	***E F***	***B F***	***A B C***	***A B C D***	
Connected glasses	789 (55.0%)	64 (64.0%)	219 (66.8%)	200 (58.8%)	154 (53.5%)	107 (45.5%)	45 (31.5%)	**<0.001**
Significant difference with other age class	–	***E F***	***D E F***	***E F***	***B F***	***A B C F***	***A B C D E***	
**What concerns you about the use of connected objects in clinical research?**								
	***N*** **= 1529**	***N*** **= 102**	***N*** **= 337**	***N*** **= 350**	***N*** **= 306**	***N*** **= 265**	***N*** **= 169**	
Protection of your personal data	574 (37.5%)	22 (21.6%)	109 (32.3%)	137 (39.1%)	128 (41.8%)	106 (40.0%)	72 (42.6%)	**0.001**
Significant difference with other age class	–	***C D E F***	***-***	***A***	***A***	***A***	***A***	
Concerns that the tool will be unreliable	543 (35.5%)	54 (52.9%)	147 (43.6%)	137 (39.1%)	96 (31.4%)	77 (29.1%)	32 (18.9%)	**<0.001**
Significant difference with other age class	–	***D E F***	***D E F***	***F***	***A B F***	***A B***	***A B C D***	
Concerns about not being able to use the object	436 (28.5%)	20 (19.6%)	75 (22.3%)	88 (25.1%)	89 (29.1%)	91 (34.3%)	73 (43.2%)	**<0.001**
Significant difference with other age class	–	***F***	***E F***	***F***	***F***	***B***	***A B C D***	
Loss of human interaction	371 (24.3%)	26 (25.5%)	70 (20.8%)	66 (18.9%)	73 (23.9%)	77 (29.1%)	59 (34.9%)	**<0.001**
Significant difference with other age class	–	–	***F***	***E F***	–	***C***	***B C***	
Ease of use	323 (21.1%)	20 (19.6%)	86 (25.5%)	87 (24.9%)	57 (18.6%)	51 (19.2%)	22 (13.0%)	**0.009**
Significant difference with other age class	–	–	***F***	***F***	–	–	***B C***	
Location tracking	285 (18.6%)	17 (16.7%)	54 (16.0%)	74 (21.1%)	57 (18.6%)	60 (22.6%)	23 (13.6%)	0.115
Significant difference with other age class	–	–	–	–	–	–	–	
Impact on understanding	234 (15.3%)	18 (17.6%)	46 (13.6%)	51 (14.6%)	47 (15.4%)	43 (16.2%)	29 (17.2%)	0.861
Significant difference with other age class	–	–	–	–	–	–	–	
Concerns of less efficient monitoring	173 (11.3%)	18 (17.6%)	37 (11.0%)	33 (9.4%)	42 (13.7%)	25 (9.4%)	18 (10.7%)	0.149
Significant difference with other age class	–	–	–	–	–	–	–	
Reluctance from healthcare professionals	124 (8.1%)	12 (11.8%)	30 (8.9%)	24 (6.9%)	22 (7.2%)	25 (9.4%)	11 (6.5%)	0.493
Significant difference with other age class	–	–	–	–	–	–	–	
**In your opinion, what would be the best study format for conducting clinical studies?**								
	***N*** **= 1529**	***N*** **= 102**	***N*** **= 337**	***N*** **= 350**	***N*** **= 306**	***N*** **= 265**	***N*** **= 169**	
Digital	276 (18.1%)	14 (13.7%)	51 (15.1%)	79 (22.6%)	60 (19.6%)	38 (14.3%)	34 (20.1%)	**<0.001**
Exclusively physical	212 (13.9%)	9 (8.8%)	31 (9.2%)	26 (7.4%)	38 (12.4%)	57 (21.5%)	51 (30.2%)	
Digital and physical	1041 (68.1%)	79 (77.5%)	255 (75.7%)	245 (70.0%)	208 (68.0%)	170 (64.2%)	84 (49.7%)	
Significant difference with other age class	–	***F***	***E F***	***E F***	***F***	***B C***	***A B C D***	
**What would be the best way to provide information regarding a clinical research protocol?**								
	***N*** **= 1529**	***N*** **= 102**	***N*** **= 337**	***N*** **= 350**	***N*** **= 306**	***N*** **= 265**	***N*** **= 169**	
Paper document with explanations from a professional	383 (25.0%)	17 (16.7%)	68 (20.2%)	64 (18.3%)	75 (24.5%)	85 (32.1%)	74 (43.8%)	**<0.001**
Video and discussion with a professional and/or a quiz on key points	378 (24.7%)	36 (35.3%)	78 (23.1%)	89 (25.4%)	77 (25.2%)	65 (24.5%)	33 (19.5%)	
A fun mobile app	768 (50.2%)	49 (48.0%)	191 (56.7%)	197 (56.3%)	154 (50.3%)	115 (43.4%)	62 (36.7%)	
Significant difference with other age class	–	***F***	***E F***	***E F***	***F***	***B C***	***A B C D***	
**If you had to provide health-related data within the context of a clinical study, what would you consider the most appropriate format?**								
	***N*** **= 1529**	***N*** **= 102**	***N*** **= 337**	***N*** **= 350**	***N*** **= 306**	***N*** **= 265**	***N*** **= 169**	
Paper questionnaire	104 (6.8%)	12 (11.8%)	20 (5.9%)	19 (5.4%)	15 (4.9%)	22 (8.3%)	16 (9.5%)	**<0.001**
Questionnaire on a mobile app	982 (64.2%)	74 (72.5%)	271 (80.4%)	268 (76.6%)	183 (59.8%)	132 (49.8%)	54 (32.0%)	
Questionnaire on a PC	443 (29.0%)	16 (15.7%)	46 (13.6%)	63 (18.0%)	108 (35.3%)	111 (41.9%)	99 (58.6%)	
Significant difference with other age class	–	***D E F***	***D E F***	***D E F***	***A B C F***	***A B C F***	***A B C D E***	
**Would you agree to share, in a secure and anonymous manner, your social media content (Facebook, Twitter, Instagram, etc.) with research staff?**								
	***N*** **= 1529**	***N*** **= 102**	***N*** **= 337**	***N*** **= 350**	***N*** **= 306**	***N*** **= 265**	***N*** **= 169**	
No	606 (39.6%)	33 (32.4%)	115 (34.1%)	128 (36.6%)	118 (38.6%)	123 (46.4%)	89 (52.7%)	**<0.001**
Yes	923 (60.4%)	69 (67.6%)	222 (65.9%)	222 (63.4%)	188 (61.4%)	142 (53.6%)	80 (47.3%)	
Significant difference with other age class	–	***F***	***E F***	***F***	***F***	***B***	***A B C D***	
**Would you be prepared to interact with an online chat system (chatbot) and send your recorded health information to a member of the research team?**								
	***N*** **= 1529**	***N*** **= 102**	***N*** **= 337**	***N*** **= 350**	***N*** **= 306**	***N*** **= 265**	***N*** **= 169**	
No	272 (17.8%)	18 (17.6%)	38 (11.3%)	47 (13.4%)	43 (14.1%)	64 (24.2%)	62 (36.7%)	**<0.001**
Yes	1257 (82.2%)	84 (82.4%)	299 (88.7%)	303 (86.6%)	263 (85.9%)	201 (75.8%)	107 (63.3%)	
Significant difference with other age class	–	***F***	***E F***	***E F***	***E F***	***B C D F***	***A B C D E***	

*Global p-value, when significant; Age classes, Significant difference with other age classes; N = number of participants per age class*.

### Focus on Food Tracking Apps

Most of participants (93.3%) declared being prepared to keep a systematic record of their food consumption using a mobile app, with the oldest agreeing less often than the youngest (*p* < 0.001). Among them, 80.5% would agree to complete such record for at least a week and 52.6% for 4 weeks or over with significant between-group difference (*p* = 0.010). Regarding the expected completion time, 38.1% of the respondents would agree to devote up to 3 min per meal, and 44.1% of the respondents would agree to devote up to 10 min per meal to such record, with no significant between-group difference (*p* = 0.070). Results and responses per age group and between-group differences are detailed in Table 6.

**Table 6:** Tracking of food consumption in clinical research by age class.

	**Total**	**18–24 years**	**25–34 years**	**35–44 years**	**45–54 years**	**55–64 years**	**65 years or over**	**Global *p*-value**
		**A**	**B**	**C**	**D**	**E**	**F**	
**Would you be prepared to keep a systematic record of your food consumption using a mobile app?**								
	***N*** **= 1529**	***N*** **= 102**	***N*** **= 337**	***N*** **= 350**	***N*** **= 306**	***N*** **= 265**	***N*** **= 169**	
No	102 (6.7%)	1 (1.0%)	7 (2.1%)	10 (2.9%)	19 (6.2%)	34 (12.8%)	31 (18.3%)	**<0.001**
Yes	1427 (93.3%)	101 (99.0%)	330 (97.9%)	340 (97.1%)	287 (93.8%)	231 (87.2%)	138 (81.7%)	
Significant difference with other age class	–	***E F***	***E F***	***E F***	***F***	***A B C***	***A B C D***	
**If yes, for how long would you be willing to complete this record?**								
	***N*** **= 1427**	***N*** **= 101**	***N*** **= 330**	***N*** **= 340**	***N*** **= 287**	***N*** **= 231**	***N*** **= 138**	
<48 h	42 (2.9%)	5 (5.0%)	7 (2.1%)	11 (3.2%)	7 (2.4%)	10 (4.3%)	2 (1.4%)	**0.010**
48 h to 4 days	91 (6.4%)	6 (5.9%)	17 (5.2%)	24 (7.1%)	13 (4.5%)	19 (8.2%)	12 (8.7%)	
4 days to 1 week	146 (10.2%)	11 (10.9%)	21 (6.4%)	34 (10.0%)	27 (9.4%)	28 (12.1%)	25 (18.1%)	
1–2 weeks	196 (13.7%)	15 (14.9%)	41 (12.4%)	52 (15.3%)	31 (10.8%)	32 (13.9%)	25 (18.1%)	
2–4 weeks	202 (14.2%)	7 (6.9%)	51 (15.5%)	50 (14.7%)	47 (16.4%)	27 (11.7%)	20 (14.5%)	
Over 4 weeks	750 (52.6%)	57 (56.4%)	193 (58.5%)	169 (49.7%)	162 (56.4%)	115 (49.8%)	54 (39.1%)	
Significant difference with other age class	–	–	***F***	–	***F***	–	***B D***	
**If yes, how much time would you be prepared to devote to this record per meal?**								
	***N*** **= 1427**	***N*** **= 101**	***N*** **= 330**	***N*** **= 340**	***N*** **= 287**	***N*** **= 231**	***N*** **= 138**	
<1 min	66 (4.6%)	4 (4.0%)	17 (5.2%)	17 (5.0%)	13 (4.5%)	11 (4.8%)	4 (2.9%)	0.070
1– 3 min	543 (38.1%)	40 (39.6%)	129 (39.1%)	148 (43.5%)	109 (38.0%)	76 (32.9%)	41 (29.7%)	
3–10 min	629 (44.1%)	40 (39.6%)	148 (44.8%)	135 (39.7%)	116 (40.4%)	112 (48.5%)	78 (56.5%)	
Over 10 min	189 (13.2%)	17 (16.8%)	36 (10.9%)	40 (11.8%)	49 (17.1%)	32 (13.9%)	15 (10.9%)	
Significant difference with other age class	–	–	–	–	–	–	–	

*Global p-value, when significant; Age classes, Significant difference with other age classes; N = number of participants per age class*.

## Discussion

### Principal Findings

By considering clinical study participants as a specific population, we confirmed their appeal for wearables and health “in pocket” technologies in research setting. This inclination to use digital tools is consistent with recent results obtained in general population (31). We also confirmed the rising trends associated with the use of smartphone apps, smart devices, and social media in medical research, which were previously reported in surveys conducted in healthcare users (3). Additionally, we were able to better understand the research participants' interest in tools of uncertain appeal, including dietary assessment tools (by considering the expected completion time and total duration), sharing of social media information, as well as chatbot conversations. Even though participants' equipment and enthusiasm seemed more important in younger age groups, our results suggest that the interest of older age classes should not be underestimated: most of these respondents expect the use of more connected tools in research practice, whatever their age. Besides, our results show that the use of paper diaries was only preferred on rare occasions, suggesting that participants recruited with the use of online tools should be provided with mobile apps over computer or paper solutions. Indeed, we identified that mobile apps are expected to provide informed consent and track health data during study conduct. Among these options, the use of a mobile app as a food diary represents a good compromise to track food intake at the touch of a finger, if its completion time does not exceed 3 min per meal. The use of mobile apps as food diaries has already been identified as a way to reduce the time to report dietary data and improve its quality (32, 33). We also noted that participants' enthusiasm toward the use of more connected tools does not go hand in hand with the conduct of remote studies. If given a choice, participants confirmed that they would rather opt for a hybrid study design, relying on both on-site and digital conduct. This can be explained, in part, by the loss of human contact which was perceived by respondents as one of their concerns in case of the use of health technologies. Our results suggest that the conduct of fully remote studies should not be considered systematically. We also identified that the protection of personal data was the most frequently reported concern, suggesting that more efforts are needed to describe risks associated with the management of individual health data and the use of digital health solutions (34).

While participants highlighted the management of their personal data as their main concern, most of the respondents would agree to share their social media content with researchers. Health information is already shared by many web users on web forums and digital platforms (35, 36). The processing of online natural language has been identified as an opportunity to enable sentiment analysis (37) or assess quality of life (38) which can be used as a complement to conventional methods of heath information tracking (39) by considering environmental, psychological and lifestyle factors (40, 41). Interestingly, even more participants confirmed that they would agree to interact with a chatbot during the conduct of clinical studies. Chatbots, while extensively used to facilitate hospital admissions and anticipate health checkup to support decision-making, are not currently used in many research studies for data acquisition purposes. Part of the research fields include mental health studies (42), including trials (43), and are also considered in behavior change studies (44), pediatric studies (45) and to evaluate how they can improve the management of chronic diseases (46, 47). Considering the importance of patient engagement in research (23, 28), we believe that their enthusiasm toward social media features and chatbots should also be leveraged to optimize retention rate throughout study participation.

### Strengths and Limitations

By conducting this survey study, we were able to obtain answers from many research participants interested in nutrition clinical studies, with an important variability in terms of age and level of education and the use of a detailed online questionnaire. To our knowledge, this is the first assessment of both equipment and expectations toward digital health and nutrition apps that was done by considering participants to nutrition clinical studies as a specific survey population. We believe that the findings of this cross-sectional survey study can serve as a starting point for repeated assessments in samples of clinical research participants to increase reproducibility in additional context and geographies. To that extent, our questionnaire is provided as Supplementary Material and may be used under Creative Commons license for non-commercial purposes (Additional File 1). While this survey mushroomed into an important dataset, comparison with prior work is however limited, as we could not identify prior research findings related to expectations toward digital heals and mobile dietary assessment tools obtained in samples of clinical research participants.

A main limitation of our study is the absence of quota-based sampling which would have facilitated further analyses in sub-groups of respondents. While feasible on a larger scale, the focus on clinical study participants as our study sample prevented the use of further screening efforts and of a larger sample of respondents. Ideally, variables including age, gender and chronic conditions could have been used to enable a quota-based sampling reflecting the French population characteristics. Another limitation of our study is the lack of information regarding the nature of chronic conditions. This information would be needed to further explore the relationship between specific indications, nutrition-related concerns, and expectations toward digital health in research settings. However, the type of chronic condition was considered as a sensitive health data for this online survey study, considering applicable data privacy regulations. Finally, the data acquisition method can also be associated with a willingness to participate in our survey study. As the respondents are participants in nutrition clinical studies who were invited to complete an online questionnaire, they can be more likely to provide study data, including nutrition information, with the use of digital tools. We therefore identified a risk of response bias when interpreting results related to the preferred way of providing health and nutrition data while taking part in a clinical study.

The successful implementation of participant-centered digital tools also relies on their endorsement by research sponsors and investigators. While our results suggest an important appeal of clinical research participant toward health technologies, the viewpoint of investigational sites personnel should also be assessed, as usage by health practitioners and may not necessarily correlate with positive assessment (11). Further work is therefore needed to continue to evaluate the drivers of successful implementation of digital health technologies based on acceptability of digital solutions by health practitioners (47). These drivers may include the availability of site staff to manage new tools and train participants and may be evaluated by conducting qualitative survey studies. Such studies may consider the opinion of both research staff and clinical study participants to identify the predictors of the implementation of digital health technologies (and associated bottlenecks) in clinical research settings.

The screening period should also be considered when interpreting our results, as enrolment was performed ahead of the COVID-19 worldwide outbreak. Despite the availability and democratization of novel health technologies in clinical practice ahead of this pandemic, some patients experienced telemedicine for the first time during this pandemic as confirmed by recent surveys (48). This surge was not only explained by concerns related to coronavirus disease, but also due to the increasing use and demand in telemedicine for behavioral health and chronic conditions to cope with the lack of conventional face to face appointments during the pandemic (49). Following the currently rising use and demand in telehealth services, we expect clinical study participants' appeal for digital health will continue to increase. Therefore, there is an opportunity to repeat our assessment regularly to better support the implementation of health technologies when designing clinical studies based on more recent results.

## Conclusion

Our study suggests that clinical study participants are keen to use various forms of digital health tools in clinical research setting, including digital health apps, food tracking apps, social media information sharing and interactions with chatbots. However, we also identified that clinical study participants remain concerned about the management of their personal data, the reliability of digital health solutions and the loss of interactions with investigators. Specific attention to ensure a high level of data security and privacy in future clinical studies should contribute to an enhanced satisfaction and trust from the participants and consequently to a higher participation rate and the collection of quality data. We believe that these results will provide supportive details for investigators and sponsors to foster the conduct of more participant-centered studies. Further work is however needed to better understand the association between interest in technologies and specific chronic conditions. Repeated assessments are suggested, as clinical research participants' propensity for technology is expected to continue to evolve following the recent surge in use and demand for innovative health services during the pandemic of COVID-19.

## Data Availability Statement

The raw data supporting the conclusions of this article will be made available by the authors, without undue reservation.

## Ethics Statement

Ethical review and approval was not required for the study on human participants in accordance with the local legislation and institutional requirements. Written informed consent for participation was not required for this study in accordance with the national legislation and the institutional requirements.

## Author Contributions

FS, LQ, MK, FC, and GF: design of the work. HC and MK: subject enrolment and data acquisition. FC: analysis. FS, LQ, MK, FC, JE, HC, and GF: interpretation of data. FS and JE: manuscript draft. FS, LQ, FC, JE, HC, and GF: revision of the manuscript. All authors contributed to the article and approved the submitted version.

## Funding

This work was funded by Danone Nutricia Research.

## Conflict of Interest

FS and LQ are the employees of Danone Nutricia Research. MK was affiliated to Danone Nutricia Research during the survey design and during data collection as an intern from the Faculty of Medicine and Pharmacy of Poitiers. GF received consultation fees from Danone Nutricia Research. Authors affiliated to Danone Nutricia Research were involved in study design, collection, analysis, interpretation of data, the writing of this article, and the decision to submit it for publication. The remaining authors declare that the research was conducted in the absence of any commercial or financial relationships that could be construed as a potential conflict of interest.

## Publisher's Note

All claims expressed in this article are solely those of the authors and do not necessarily represent those of their affiliated organizations, or those of the publisher, the editors and the reviewers. Any product that may be evaluated in this article, or claim that may be made by its manufacturer, is not guaranteed or endorsed by the publisher.
