# Supplementary material for: Attitudes and Expectations of Clinical Research Participants Toward Digital Health and Mobile Dietary Assessment Tools: Cross-Sectional Survey Study
Source: Front Digit Health. 2022 Mar 9;4:794908. doi: 10.3389/fdgth.2022.794908 (PMC8959345; doi:10.3389/fdgth.2022.794908)
Supplement: Supplementary file 1 [file Data_Sheet_1.docx]

**Survey on connected health/nutrition record acceptance**

Dear Sir/Madam,

As part of a survey organised by Danone Research and in partnership with your research centre, we are inviting you to share your opinion on health-related applications and connected objects.

Survey justification

Many studies involve keeping an accurate record of food consumption, physical activity or other health information. Recent technological advances are opening up new avenues in this regard. The use of smartphones or connected objects is becoming increasingly common in our everyday lives and offers numerous possibilities for clinical research. The purpose of this survey is to assess a clinical study participant’s acceptance of this technology.

Participation criteria

To complete this survey, the only requirement is that you must be at least 18 years of age and be currently participating or have previously participated in a clinical study.

Survey procedure

You will be asked 27 questions. It is estimated that the survey will take less than 5 minutes to complete.

Data confidentiality

This survey does not require you to provide any personal data. The information you provide will be analysed in relation to the objectives set out above.

After reading this document you may complete the questionnaire

**Part I: Socio-demographic data**

1. Your age:

- 18-24 years
- 25-34 years
- 35-44 years
- 45-54 years
- 55-64 years
- 65 years or over

1.b) Sex:

- Female
- Male
- Other

1. Your place of residence:

- Village (less than 2000 inhabitants)
- Small city (2000-10,000 inhabitants)
- Medium-sized city (10,000-50,000 inhabitants)
- Large city (over 50,000 inhabitants)

1. Level of education:

- No formal education
- Primary education
- Lower secondary education
- Secondary education (Upper secondary, Technical or vocational qualification)
- Third level (Primary Degree, Professional, Qualification, Post graduate certificate or Diploma, Postgraduate Degree, Doctorate (PhD)

3.b) Socio-professional category:

- Farmers
- Craftspeople, retail traders, business owners
- Senior management and professionals
- Middle management
- Skilled employees
- Unskilled employees
- Skilled workers
- Unskilled workers
- Unemployed
- Student
- Retired

1. Do you suffer from a chronic condition? Yes No

**Part II: Smartphones and connected objects**

1. Do you own a smartphone? Yes No

*If No, skip to question 2*

If yes, how many health-related mobile apps do you use? (Nutrition, physical activity, weight, sleep, health coaching, well-being, meditation, etc.)

- None
- One
- Two or three
- Four or more

If you use at least one health-related mobile app, please specify how often you use it

- Less than once a month
- 1 to 3 times a month
- Once a week
- 2 to 5 times a week
- At least once a day

On average, how much time do you spend on these health apps per use?

- Less than 1 minute
- 1 to 5 minutes
- Over 5 minutes

1. What are your main expectations from mobile health apps ?

- Monitoring your energy intake
- Weight loss monitoring
- Weight gain monitoring
- Weight maintenance monitoring
- Assessing the quality of your food
- Maintaining/improving your health
- Assessing of your physical activity
- Monitoring a chronic condition
- Monitoring your sleep
- A meditation tool
- Period or pregnancy tracking
- No expectations
- Other: __________________________

1. What are your main concerns about using health-related mobile apps ?

- Not useful
- Protection of your personal data
- Location tracking
- Lack of reliability
- Too time-consuming
- Poorly designed interface
- Does not meet your needs
- The frequency of adverts
- The need to create a personal account
- Other: __________________________

1. Do you use a connected watch or wristband ? Yes No

*If No, skip to question 5*

If yes, why? (2 answers maximum)

- Assessing of your physical activity
- Sports reasons
- Weight loss monitoring
- Cardiovascular monitoring
- Improving/maintaining your health
- Monitoring a chronic condition
- Monitoring your sleep
- Because of the device’s appearance
- To receive smartphone notifications

1. What concerns you about using a connected watch or wristband ?

- Not useful
- Protection of your personal data
- Location tracking
- Battery life
- Appearance
- Weight
- Discomfort
- Price
- Other: __________________________

**Part III: Clinical studies**

1. If you had to provide health-related data within the context of a clinical study, what would you consider to be the most appropriate format?

- Paper questionnaire
- Questionnaire on a mobile app
- Questionnaire on a PC

1. As part of a clinical study, would you be prepared to keep a systematic record of your food consumption using a mobile app? Yes No

*If No, skip to question 3*

If yes, for how long would you be willing to complete this record?

- Less than 48 hours
- 48 hours to 4 days
- 4 days to 1 week
- 1 to 2 weeks
- 2 to 4 weeks
- Over 4 weeks

How much time would you be prepared to devote to this record per meal?

- Less than 1 minute
- 1 to 3 minutes
- 3 to 10 minutes
- Over 10 minutes

1. Would you be willing to use a health-related connected object within the context of a clinical study? Yes No

*If No, skip to question 4*

If yes, please specify which one(s)

Connected weighing scales Yes No

Connected plate Yes No

Connected watch/wristband Yes No

Connected glasses Yes No

Connected patch Yes No

1. What concerns you about the use of connected objects in clinical research?

- Protection of your personal data
- Location tracking
- Loss of human interaction
- Concerns of less efficient monitoring
- Impact on understanding
- Reluctance from healthcare professionals
- Ease of use
- Concerns about not being able to use the object
- Concerns that the tool will be unreliable
- Other: __________________________

**Part IV: The future of clinical research**

1. Clinical studies evolve with technological progress. In your opinion, what would be the best study format for conducting clinical studies?

- Digital (monitoring via videoconferencing only)
- Exclusively physical (visiting a professional for each measurement/reading)
- Digital and physical

1. What concerns you about the use of videoconferencing in clinical research?

- Loss of human interaction
- Concerns of less efficient monitoring
- Impact on understanding
- Reluctance from healthcare professionals
- Ease of use
- Your lack of access to a digital tool
- Concerns about not being able to use the digital tool
- Concerns that the tool will be unreliable
- Protection of your personal data
- Other: __________________________

1. In your opinion, what would be the best way to provide information regarding a clinical research protocol?

- Paper document with explanations from a professional
- Video and discussion with a professional and/or a quiz on key points
- A fun mobile app

1. Within the context of a clinical study, would you agree to share, in a secure and anonymous manner, your social media content (Facebook, Twitter, Instagram, etc.) with research staff?

Yes No

1. Within the context of a clinical study, would you be prepared to interact with an online chat system (chatbot) and send your recorded health information to a member of the research team?

Yes No

1. Do you have any comments or points you feel are important to raise regarding connected health, data in relation to connected objects, or the approach taken to health through new technologies?

____________________________________________________________________________________________________________________________________________________________________________________________________________________________________________________________________________________________________________________________________________________________________________________________________________________________________________________________________________________
